# Correlation of Computed Tomography with Pathological Features in Angiomatous Nasal Polyps

**DOI:** 10.1371/journal.pone.0053306

**Published:** 2012-12-31

**Authors:** Li-Bo Dai, Shui-Hong Zhou, Ling-Xiang Ruan, Zhou-Jun Zheng

**Affiliations:** 1 Department of Otolaryngology, The First Affiliated Hospital, College of Medicine, Zhejiang University, Hangzhou, Zhejiang Province, China; 2 Department of Radiology, The First Affiliated Hospital, College of Medicine, Zhejiang University, Hangzhou, Zhejiang Province, China; 3 Department of Pathology, The First Affiliated Hospital, College of Medicine, Zhejiang University, Hangzhou, Zhejiang Province, China; University of Navarra, Spain

## Abstract

**Background:**

Angiomatous nasal polyps (ANPs), also known as angiectatic polyps, have rarely been reported in the literature. ANPs are characterized by extensive vascular proliferation and ectasia. ANPs can grow rapidly and exhibit aggressive clinical behavior that could simulate malignancy preoperatively, and they are easily confused with other diseases. In the present study, we analyzed the correlation between the computed tomography (CT) findings of nasal angiomatous polyps and their pathological features.

**Methods:**

We evaluated CT findings and pathological features of 31 surgically proven ANPs.

**Results:**

The study population included 16 males and 15 females aged between 27 and 81 years (mean age, 53.5 years). On CT, the masses were heterogeneous; they had a soft tissue density and filled the maxillary and/or nasal cavities. Calcifications were found in 2 of the 31 cases. The lesions showed a clear boundary (15/31). The low**-**density shading on CT was related to the inflammatory, necrotic, and cystic changes, and the high**-**density shading on CT was related to hemorrhagic areas of the mass. On contrast-enhanced CT, the center of the lesions was non-enhanced with peripheral intensification due to occlusion or compression of feeder vessels of the polyp center, and the inflammatory cells and neovascularization around the edge of the mass. The most common site of maxillary wall erosion was the medial wall (21/31), followed by the posterior lateral wall (3/31), upper wall (2/31), and septum (3/31). Of these, the nasal cavity and/or maxillary sinus were enlarged in 28 cases. These findings were associated with the chronic progress of nasal angiomatous changes.

**Conclusions:**

CT of ANPs may demonstrate benign bone changes associated with the lesions and may also reflect the fact that ANPs do not invade peripheral soft tissue. CT demonstrated these lesions consistently and provided information useful for surgical planning.

## Introduction

Based on the predominant elements seen on histological evaluation, inflammatory or allergic sinonasal polyps (SNPs) have been classified into five types: edematous, glandular, fibrous, cystic, and angiectatic or angiomatous. Angiomatous nasal polyps (ANPs) are rare, representing only 4–5% of all SNPs [1]. ANPs can grow rapidly and exhibit an aggressive clinical behavior that can simulate malignancy preoperatively^1^. The clinical and radiological characteristics of these lesions have considerable potential for confusion with neoplastic processes, including juvenile angiofibroma, inverted papilloma, and hemangioma. To our knowledge, three correlative clinical studies have reported on the relationship between radiological findings and the pathological features of ANPs [2]–[4]. Although reports of CT findings of sinonasal ANPs have been published and given that ANP imaging is reported to be rather nonspecific, it would be useful to perform a comprehensive systematic analysis of a large group of lesions to determine whether imaging features specific for ANPs can be identified.

In the present paper, we present a retrospective analysis of the relationship between computed tomography (CT) findings and clinicopathological features of 31 patients with ANPs between January, 1997 and August, 2011.

## Results

### Clinical Characteristics

There were 16 males and 15 females aged between 27 and 81 years (mean, 53.5 years). The history of symptoms ranged from 1 month to 5 years. Clinical symptoms contributing to the diagnosis were varied and nonspecific, including unilateral nasal obstruction and epistaxis (Table 1). Endoscopy revealed purple soft masses alternating with black necrotic areas in the middle nasal meatus in 25 cases that bled easily on touching. The mass extended into the choana and nasopharynx in three cases. Lund–Kennedy (LK) system scores ranged from 0 to ∼16 (mean, 2.54±0.56; Table 2).

**Table 1:** The demographic and clinical characteristics of 31 angiomatous nasal polyps.

Characteristic	Number (%)
**Age**	
Mean	53.5±11.5
>70	5(16.1)
40∼70	16(51.6)
<40	10(32.3)
**Sex**	
Male	16(51.6)
Female	15(48.4)
**Clinical symptoms**	
only unilateral NO	7(22.6)
only unilateral NB	5(16.1)
unilateral NO +NB	11(35.5)
unilateral NO +ND	4(12.9)
ophthalmoptosis	3(9.7)
facial swelling and pain	1(3.2)
bilateral nasal obstruction	3(9.7)
**Past history**	
snare resection	6(19.4)
Caldwell-Luc operation	1(3.2)
tobacco use	10(32.5)
alcohol abuse+ tobacco use	5(16.1)
hypertension	5(16.1)

Note: NO: nasal obstruction; NB: nasal bleeding; ND: nasal discharge.

**Table 2:** Number and Percentage of Patients Having a Particular Nasal Endoscopy Sign and the Average Lund-Kennedy (LK) Score (and Ranges) for Each Sign.

Nasal Endsocopy Sign	Number (%)	Average Score	Range of Scores
Discharge	4(12.9)	2.10±0.78	0–3
Edema	20(64.5)	0.57±0.10	0–2
Polyp	25(80.6)	1.89±0.34	0–6
Scarring/adhesions	6(19.4)	0.71±0.21	0–2
Crusting	3(9.7)	0.68±0.11	0–2
None	2(6.5)	0	0–0
Total	29(93.5)	2.54±0.56	0–16

### CT Findings

#### 1. Site, extent, and density of lesions (Figs. 1, 2, 3, 4)

The lesions originated on the left and right sides in 18 and 13 cases, respectively. The scores using the Lund-Mackay (LM) scale were from 0 to approximately 20 (mean, 6.23±0.76; Table 3). The most common site of tumor origin was the maxillary sinus (24/31, 77.4%), and less frequently the nasal cavity (22.6%). On CT, the masses were heterogeneous; they had a soft tissue density and filled the maxillary and/or nasal cavity. CT values ranged from 23.0 to 56.0 HU. Of 24 masses located in the maxillary sinus, 21 involved the ipsilateral nasal cavity. Of these, three cases involved the choana, two extended into the orbit, 17 involved the ipsilateral ethmoid sinus, and three involved the contralateral nasal cavity. ANPs in the nasal cavity partially involved the juncture of the maxillary sinus and nasal cavity. Calcifications were found in 2 of the 31 cases. Twenty-nine of 31 (93.5%) lesions displayed sharply defined margins and two of 31 (6.5%) lesions were poorly defined. When the fat space between lesions and surrounding tissues was evident because the lesions were too large, we deemed that the lesions had well-circumscribed margins. Conversely, when the fat space between lesions and surrounding tissues was not evident–a finding that caused the fat space to tightly contact surrounding muscles and caused the lesion density and contact boundary to be indistinguishable–the lesion was deemed poorly defined. We confirmed lesions to be primary or secondary in the maxillary sinus or in the nasal cavity mainly according to the site of the main body of the lesion. Six cases with contrast enhancement showed minimal enhancement at the edges of the lesions. None of the cases showed invasion into the sphenoid sinus (Table 4).

**Table 3:** CT and average Lund-MacKay (LM) Score.

Sinus CT Scan Region	Number (%)	Average LM Score	Range of Scores
Maxillary	24(77.4)	2.23±0.59	0–4
Frontal	4(12.9)	1.01±0.33	0–2
Anterior ethmoid	14(45.2)	2.87±0.78	0–4
Posterior ethmoid	3(9.7)	1.67±0.43	0–3
Sphenoid	0	/	/
Ostiomeatal complex	16(51.6)	2.55±0.98	0–4
Total	31(100.0)	6.23±0.76	0–20

**Table 4:** CT features of 31 angiomatous nasal polyps.

Features		Number (%)
Site		
Nasal cavity		7(22.6)
Maxillary sinus		24(77.4)
Calcification		2(6.5)
Bony changes		31(100.0)
wall erosion andabsorption	Maxillary medial wall	21(67.7)
	Maxillary posterior lateral wall	3(9.7)
	Maxillary upper wall	2(6.5)
Enlarged nasal cavity and/or maxillary		28(90.3)
Bony sclerosis		25(80.6)
	Maxillary posterior lateral wall	20(64.5)
	Maxillary upper wall	5(16.1)
Contrast enhancement		6(19.4)
	minimal enhancement in edge of the lesions	5(5/6)
	No enhancement in edge of the lesions	1(1/6)
	No enhancement in centre of the lesions	6(6/6)

**Figure 1:**
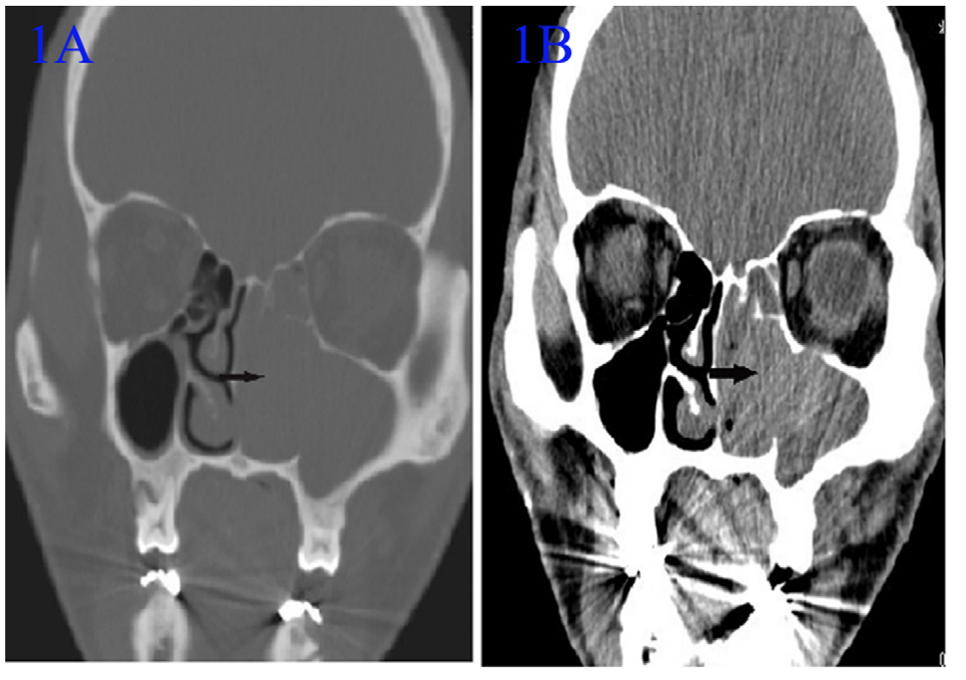
Computed tomography (CT) of a recurrent angiomatous polyp (ANP) in the left maxillary sinus 10 years after ANP removal surgery. CT showed an irregular, soft-tissue mass in the left maxillary sinus. The density was heterogeneous, and the CT value was approximately 45 HU. The medial maxillary sinus wall was completely destroyed (arrow). The mass involved the left nasal cavity and the left ethmoid sinus. A: noncontrast coronal CT in bone window; B: noncontrast coronal CT in soft-tissue window.

**Figure 2:**
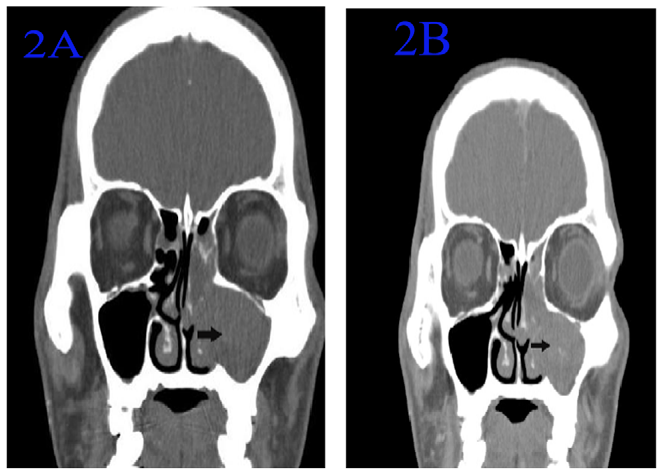
CT of an ANP in the left maxillary sinus involving the ipsilateral ethmoid sinus. 2A: Noncontrast coronal CT in soft-tissue window showed that a high-density mass filled the total left maxillary sinus. The density of the mass was homogeneous. The left maxillary sinus was expansile, and the medial maxillary sinus wall was partially destroyed (arrow). The mass involved the ipsilateral ethmoid sinus. 2B: Contrast-enhanced CT: The mass was non-enhanced.

**Figure 3:**
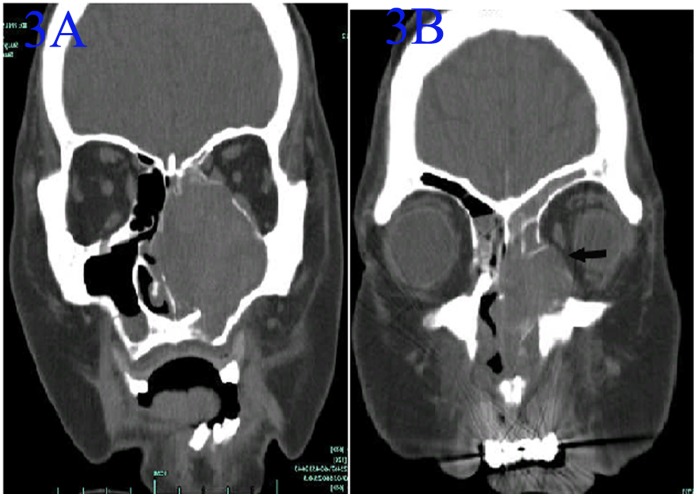
CT of an ANP in the left maxillary sinus involving the ipsilateral orbit. A :Noncontrast coronal CT in soft-tissue e window showed that a high-density mass filled the total left maxillary sinus. 3B: Noncontrast coronal CT in soft-tissue e window showed that the lesion was involved in the left orbitand the eyeball shifted laterally (arrow).

**Figure 4:**
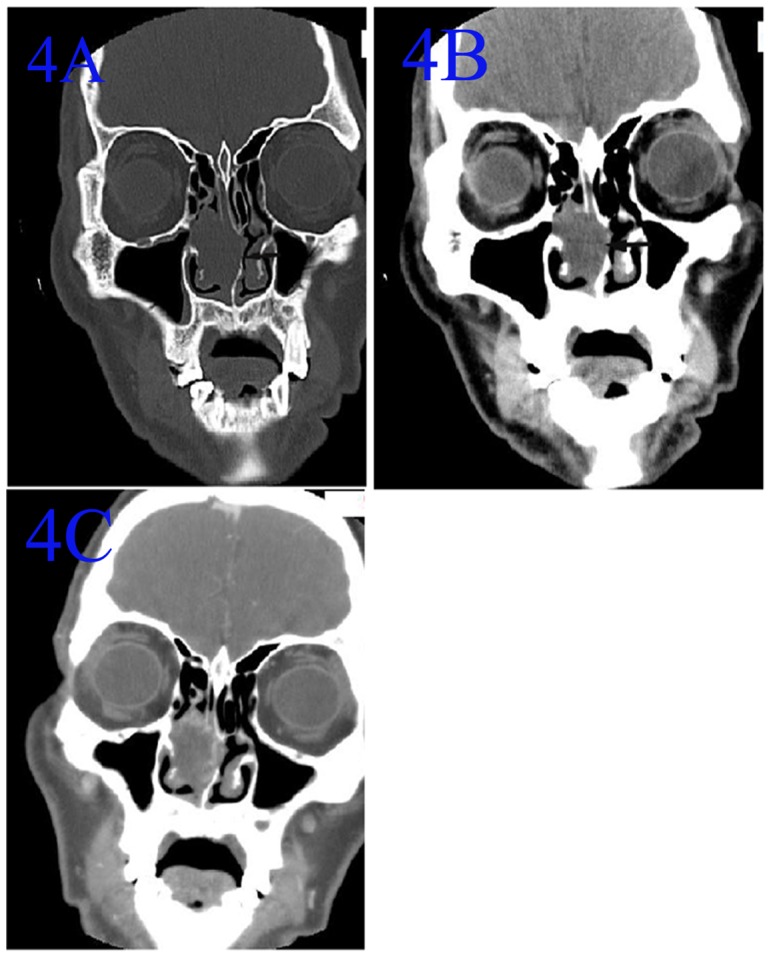
CT of an ANP confined within the right nasal cavity. Noncontrast coronal CT in bone window(A) and in soft-tissue e window(B) showed a soft-tissue mass limited within the right nasal cavity. The edge was irregular, and the nasal septum was compressed and deformed (arrow). The CT value was 26 HU. The walls of the right nasal cavity were intact. The maxillary and ethmoid sinuses were not involved. 4C: Contrast-enhanced CT showed the lesion was non-enhanced.

#### 2. Bony changes

All of the lesions caused changes in the adjacent bone, including expansile remodeling, hyperemic demineralization/resorption, frank osseous erosion, and hyperostosis of the maxillary walls. Hyperostosis seems much more likely to be related to chronic post-obstructive sinus disease, because it occurred along the posterior and lateral maxillary sinus walls and the tumors were located along the medial sinus wall and nasal cavity. The most common site of maxillary wall erosion was the medial wall (21/31)followed by the posterior lateral wall (3/31), upper wall (2/31). Of these, the nasal cavity and/or maxillary sinus were enlarged in 28 cases. Bony sclerosis was most evident along the posterior lateral wall (20/31), followed by the upper wall (5/31; Table 4).

#### 3. Diagnosis accuracy of preoperative CT

Of the total of 31 cases, eight were diagnosed as ANPs, two as nasal polyps, and two as fungal rhinosinusitis; 10 cases were considered benign tumors and eight were misdiagnosed as malignant tumors.

**Figure 5:**
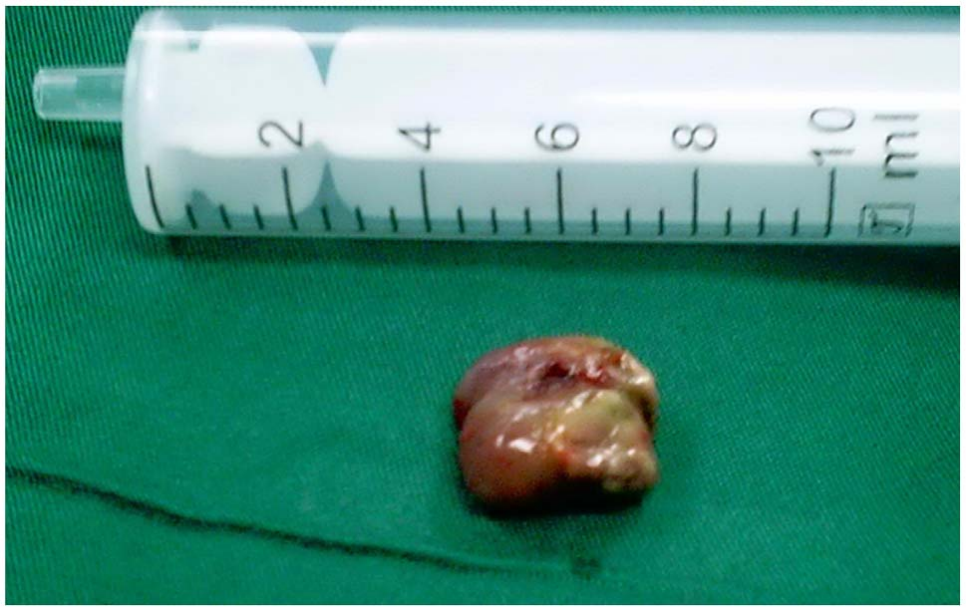
Grossly, the tumors were purple, necrotic masses.

### Surgical Findings

The results of surgical evaluation of 31 lesions showed a good correlation with those of CT. Of the 31 cases included in this study, the lesions were limited to the maxillary sinus in three cases and involved the maxillary sinus and nasal cavity in 21 cases, the choana in three, and the orbit in two. The lesions involved the septum and affected the contralateral nasal cavity in three cases and were limited to within the nasal cavity in seven. Intraoperatively, wall erosion was found most frequently in the lateral walls of the nasal cavity (21/31), followed by the posterior lateral walls (3/31) and upper walls (2/31). We also found that the tumors were purple soft masses alternating with black necrotic areas and that they bled easily after touching. However, no significant hemorrhaging occurred during surgery.

### Pathological Findings (Figs. 5, 6, 7, 8 *)*

Grossly, the tumors were purple necrotic masses. On sections, the cut surfaces were firm, with glistening tan areas, alternating with yellow-brown and hemorrhagic necrotic zones that showed cystic degeneration. The microscopic findings were similar in all cases. The surface of the tumors was covered with pseudostratified ciliated columnar epithelium or stratified squamous epithelium having edematous stroma, with infiltration by many inflammatory cells. The lesions were filled with clusters of irregularly shaped, thin-walled blood vessels (31/31) and showed thrombus formation (29/31), which was associated with ischemic necrosis. These thin-walled vessels were embedded in a large pool of amorphous material, and the stroma also showed an abundance of collagen (15/31) and calcification (3/31). Hemosiderin-laden macrophages were present due to recent hemorrhages.

**Figure 6:**
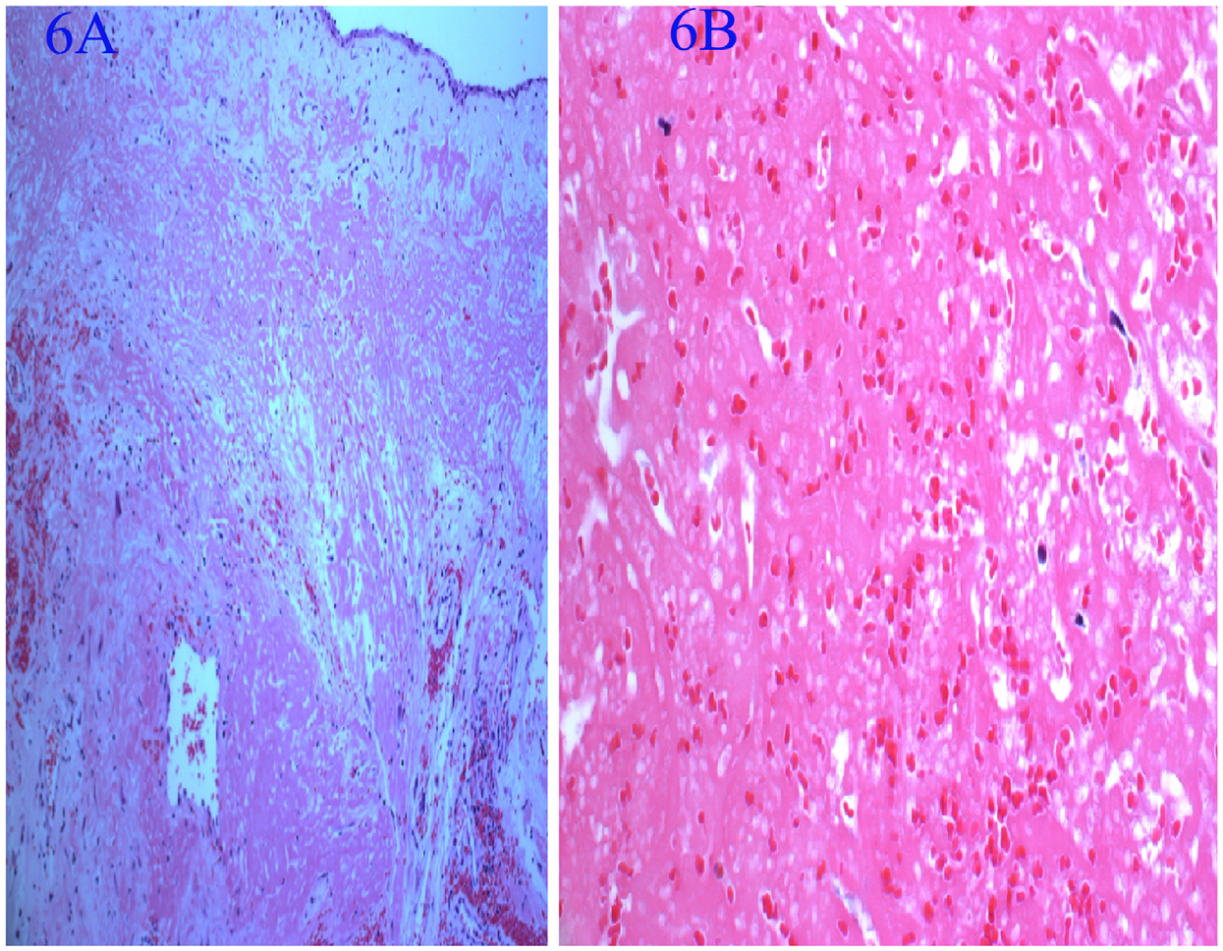
Pathological findings. A: Light microscopy showed the tumor covered with pseudostratified, ciliated, columnar epithelium or stratified squamous epithelium with edematous stroma, and many infiltrating inflammatory cells (hematoxylin and eosin staining; original magnification, ×100). B: Proliferation of thin-walled vascular channels. Separated blood vessels exhibited concentric accumulation of amorphous material (hematoxylin and eosin staining; original magnification, ×100).

**Figure 7:**
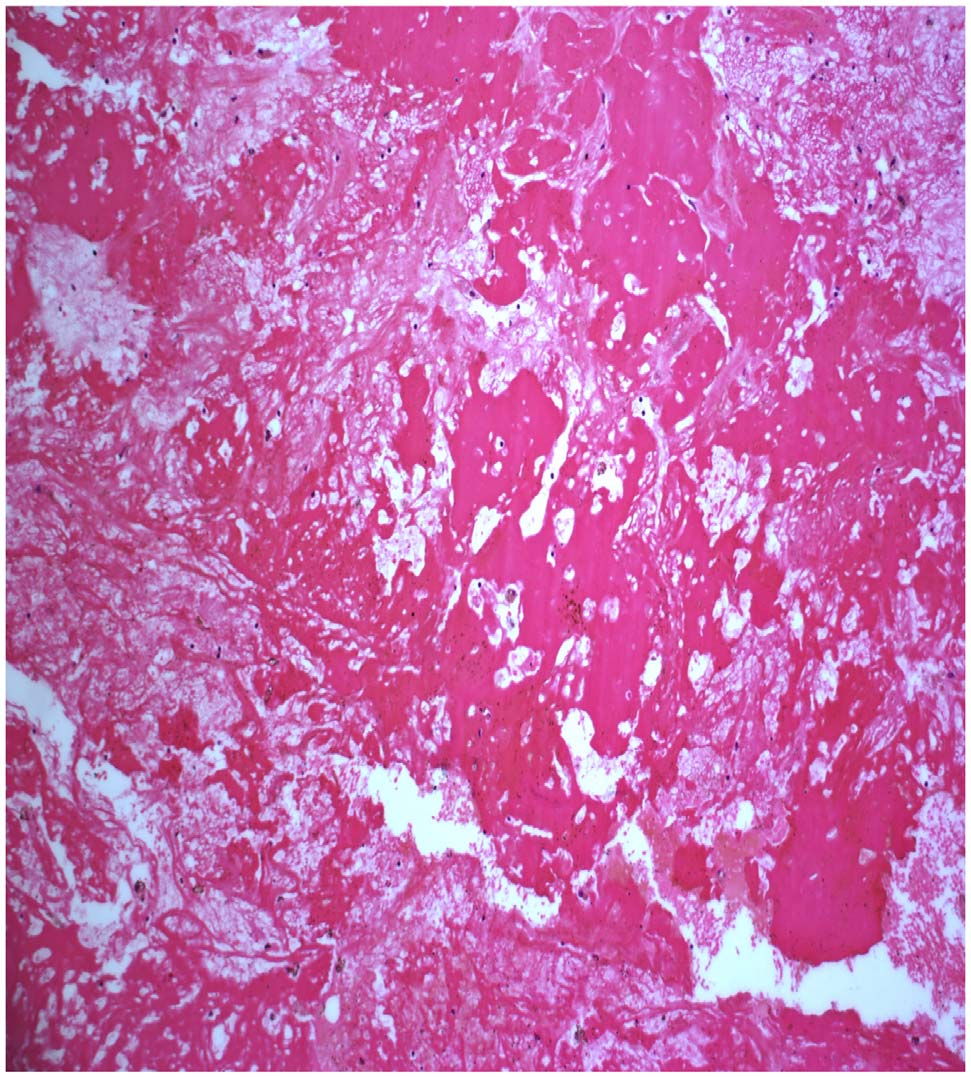
Pathological finding of edematous stroma and thrombus formation (hematoxylin and eosin staining; original magnification, ×100).

**Figure 8:**
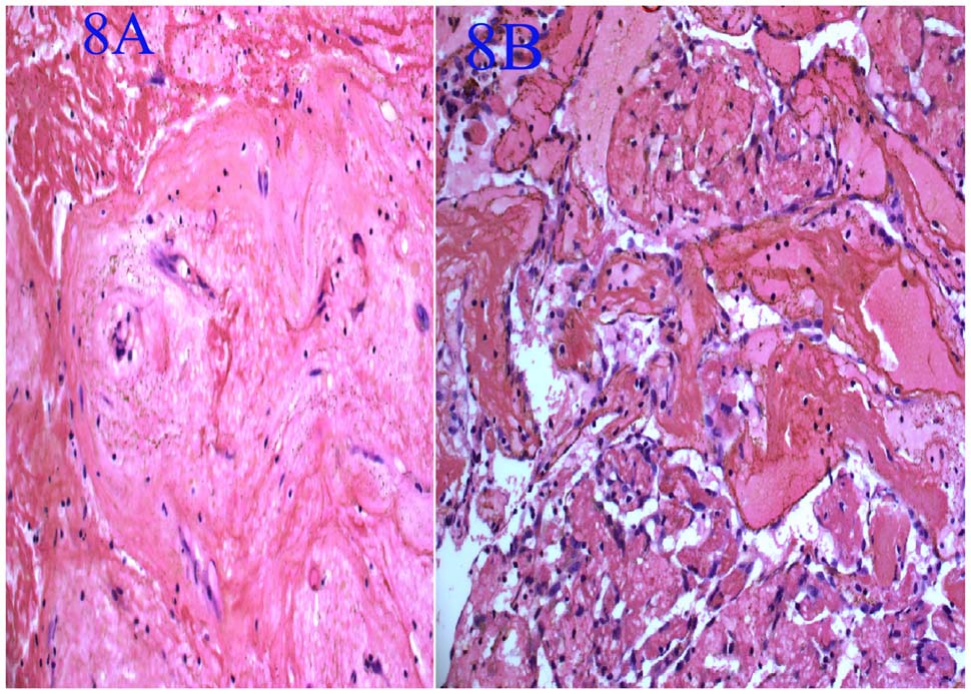
Pathological findings of an earlier organized thrombus (8A: hematoxylin and eosin staining; original magnification, ×400) and organized thrombus (8B: hematoxylin and eosin staining; original magnification, ×200).

### Follow-up

Only one patient showed disease recurrence 10 years after surgery. A repeat operation was performed, and the patient remains well after 48 months. Other patients were followed-up from 3 months to 11 years (mean follow-up period, 60.1 months), and no incidences of recurrence were observed in this study population.

## Discussion

Our data suggest that CT findings are associated with pathological features.

### Correlation between CT Findings and Pathological Features of ANPs

Regardless of the site of origin, the tumors in our cases demonstrated several common clinical and radiological characteristics: the patients’ chief symptoms included persistent epistaxis and nasal obstruction. Moreover, tumors were sufficiently large to cause significant bone erosion, and the pattern of enhancement on CT was non-homogeneous.

#### 1. Density and enhancement findings were consistent the pathological features

On CT, the mass showed heterogeneous density filling the nasal cavity and/or maxillary sinus. The CT value ranged from 23.0 to 56.0 HU, and the mass showed minimal enhancement at the edge of the lesions. These findings on CT were correlated with the mixture of the extensive areas of hemorrhage, organized thrombi, necrosis, and inflammatory cells in this part of the polyp. The inflammatory, necrotic, and cystic tissues were responsible for the low density of the mass on CT, and the high density of the mass was due to hemorrhagic lesions. The center part of the polyp showed no enhancement and only minimally peripheral enhancement, as observed for a simple polyp [3]. Although all cases were filled with clusters of irregularly shaped, thin-walled blood vessels and extensive vascular proliferation and ectasia, the dilated vessels in the center were slow due to thrombus formation and the substantial infarction responsible for the lack of enhancement. Fibrous collagenous tissues in the lesions occassionally caused enhancement on CT; however, this event usually occurred in the later period of contrast-enhanced CT. Therefore, if the scan period is inappropriate, CT would not indicate such enhancement. Because the peripheral area of the mass showed inflammation and vascular proliferation, the blood supply was more abundant than at the center of the mass, leading to enhancement in the peripheral area. Calcium salt deposition was observed in organized thrombi and necrotic areas, leading to punctuate calcification on CT.

#### 2. Bony changes were consistent with the pathological features

The lesions in all cases caused changes in the adjacent bones, including destruction, compression, and hyperostosis of maxillary walls. The most common site of maxillary wall erosion was the medial wall (21/31). The nasal cavity and/or maxillary sinus were enlarged in 28 cases. These CT findings were correlated with the chronic development of ANPs and were confirmed by surgery. These observations suggest that preoperative CT could provide an accurate basis for determination of the surgical scope. Bone resorption and neogenesis are also associated with pathological features such as long-term chronic paranasal sinusitis and active osteoblast growth. These CT features, in conjunction with the associated bone erosion, make diagnosis based on CT difficult [1]. However, the masses on CT showed clear and smooth edges and did not invade the peripheral soft tissue, which was associated with a non-tumor growth pattern of ANPs. On CT, ANPs do not usually invade the pterygopalatine fossa or sphenoid sinus [1], [2], [6], [7], [8].

### Limitations of the Present Study

Although our findings suggest that CT may aid the diagnosis of ANPS, it lacked MRI information. MRI is now the investigation of choice for soft-tissue lesions of the nasal cavity, including ANPs [3], [4].

### Naming of ANPs

The naming of ANPs is very chaotic; names include inflammatory granuloma telangiectaticum, vascular granuloma, pseudo-angioma, hemorrhage necrotic polyp, and angiomatous polyp. We feel that the term angiomatous polyp is appropriate because it reflects the fact that the lesion is not a real tumor and that the mass is clinically characterized by hemorrhage and chronic proliferation.

### Pathogenesis of ANPs

The true pathogenesis of ANPs is still not clear, although many hypotheses have been proposed. One is that inflammation and/or allergy of the maxillary sinus and nasal cavity cause proliferation, dilation, rupture, and hemorrhage of mucosal blood vessels, leading to mucosal edema and polypoid changes [1]. In addition, Som concluded that the angiomatous polyp is a fibrosed and vascularized nasal and/or nasopharyngeal mass, presumably originating as a response to minor trauma [6]. Another hypothesis is that based on the existence of the maxillary sinus and/or nasal cavity polyp, the polyp pedicle is compressed, resulting in stasis, ischemia, and necrosis of the polyp. Batsakis identified four extraantral sites of vulnerability to vascular compromise for antrochoanal polyps: the ostial exit site, the posterior end of the inferior turbinate, the posterior choana, and the most dependent part within the nasopharynx [7]. Compression of blood vessels in these areas is hypothesized to result in initial vascular dilatation and stasis, and extravascular edema. This leads to venous infarction followed by neovascularization of the polyp, setting the stage for repeat vascular occlusion and infarction [7]. Extensive extravasation of blood components through thin-walled vessels results in areas of hemorrhage and accumulation of large perivascular pools of amorphous eosinophilic material [2]. We support this theory. The MR imaging findings reported by De Vuysere [3] and Wang [4], correspond with the angiomatous polyp as reported by Batsakis [7]. The maxillary sinus first had the hemangioma itself, and was then involved by the polyp, which grew inward [8].

### Differential Diagnosis

Based on our previous experience, in which CT findings of ANPs were correlated with the pathological features and surgical findings, a CT study appears effective for a presurgical diagnosis. Our experience suggests that ANPs can cause substantial bone erosion, the appearance of which on CT may mislead the radiologist to make a false diagnosis of a malignant tumor. This type of bone erosion can also occur with other benign lesions, such as inverted papilloma, juvenile angiofibroma, and hemangioma. The radiologist should not presume that a lesion is malignant simply because a defect in the bone is visualized on imaging studies (Table 5) [1], [5], [6], [9]–[15].

**Table 5:** Clinical and CT imaging features in the angiomtous nasal polyps and diverse entities.

Entities	Age/sex	Clinical features	origin	CT findings
Angiomatous nasal polyps	A wide age range, no gender predominance	varied and nonspecific, including unilateral nasal obstruction and epistaxis	Maxillary sinus, nasal cavity	A soft tissue mass extending from the maxillary sinus/nasal cavity. Isolated expansile nasal vault masses without a nasopharyngeal mass or nasal-nasopharyngeal masses of considerable size that did not invade either the pterygopalatine fossa or the sphenoid sinus. No enhancement or minimal enhancement in edge of the lesions. The edge of ANPs on CT is clear and does not invade the peripheral fat layer.
Juvenile angiofibroma	only in youngmales	The typical clinical symptoms are nasal obstruction and recurrent epistaxis.	posterior nasal fossa, close to the sphenopalatine foramen	an extension of the tumor into the pterygopalatine fossa This causes a widening of this space, with anterior bowing of the posterior antral wall, extension through the roof of the nasopharynx into the sphenoid sinus, and enhanced lesions on contrast CT scans
Vascular tumors	No sex, gender predominance	unilateral nasal epistaxis, or/and obstruction	Most in the anterior nasal septum	a soft tissue density mass may cause bone remodeling and destruction, greater enhancement on contrast-CT than ANPs
Nasal inverted papilloma	most commonlyin the 6th to 8thdecade of life.male preponderance	unilateral nasal epistaxis,obstruction, recurrent, a potentialfor malignancy. There is anassociation with synchronouscancer in 10%	the most commonsite of origin is thelateral nasal wall	Homogeneous soft tissue mass; it has a density like that of soft tissue and may contain calcium. Focal bone remodeling and sclerosis are also frequently seen. The mass shows heterogeneous enhancement after injection of contrast material.
Non-invasive fungal rhinosinusitis	an increased incidence inelderly females	nasal stuffiness, bloody discharge	Most common site is maxillary	The presence of diffuse increased attenuation within the paranasal sinuses and nasal cavity should be considered as chronic allergic hypersensitivity aspergillosis, along mottled hyperdense foci of variable size. Bony destruction associated with fungal infection is rare.
Malignant tumor	Peak incidenceis in the sixth andseventh decades,with a malepredominance	nasal obstruction with epistaxys,with a relatively short diseasehistory	the maxillary sinus and the nasal cavity as the most common sites of origin	The bony erosion of malignant tumors is destructive and the edges are indistinct; moreover, the peripheral fat layer is invaded and disappears. On a contrast-CT scan, malignant tumors show heterogeneous enhancement, but hyperostosis and removal of maxillary/nasal cavity walls are rare

#### 1. Juvenile angiofibroma

Preoperatively, ANPs are often confused with juvenile nasopharyngeal angiofibromas [6], [11]. With extremely rare exceptions, nasopharyngeal angiofibromas occur only in young males^1^. The possibility of ANPs should always be considered before a diagnosis of nasopharyngeal angiofibroma is considered in elderly or female patients [1]. On CT, ANPs were either non- or minimally enhanced lesions, but more importantly, they were either isolated expansile nasal vault masses without a nasopharyngeal mass or nasal-nasopharyngeal masses of considerable size that did not invade either the pterygopalatine fossa or the sphenoid sinus [5]. Radiographic findings specific to juvenile nasopharyngeal angiofibroma, such as tumors arising in the nasopharynx or in the sphenopalatine foramen [10], are an extension of the tumor into the pterygopalatine fossa. This causes a widening of this space, with anterior bowing of the posterior antral wall [11], extension through the roof of the nasopharynx into the sphenoid sinus [11], and enhanced lesions on contrast CT scans [1].

Thus, the appearance of an expansile nasal tumor without a nasopharyngeal mass virtually excludes a diagnosis of angiofibroma. Similarly, any sizable nasopharyngeal mass that has grown sufficiently forward to involve and expand the posterior nasal vault and that does not extend into the pterygopalatine fossa or sphenoid sinus is extremely uncharacteristic of the growth pattern of an angiofibroma [1].

#### 2. Vascular tumors

Vascular tumors are the most common type of nonepithelial tumor of the nasal cavity and nasopharynx [1], and the prominent vascular component in ANPs can pose problems with regard to differential diagnosis. The ANPs should be mainly differentiated from capillary or cavernous hemangiomas. Angiomas are composed of irregular vascular channels lined with benign-appearing, usually flattened endothelial cells embedded in edematous stroma. Sinonasal angiomas present with nasal obstruction and epistaxis, and show no sex or age predominance [1]. Most nasal hemangiomas arise from the nasal septum or vestibule and are of the capillary type. Only a few arise from the lateral wall of the nose, and these are usually cavernous. In the paranasal sinuses, hemangiomas are even rarer [12]. On a CT scan, sinonasal angiomas show greater enhancement on contrast-CT than ANPs.

#### 3. Nasal inverted papilloma

As clinical symptoms are nonspecific, the diagnosis of an inverted papilloma can be difficult to establish and is sometimes missed. Excluding inverted papilloma as a diagnosis was not possible based on clinical or radiological grounds alone [2]. On CT, an inverted papilloma is homogeneous; it has a density like that of soft tissue and may contain calcium. The mass shows heterogeneous enhancement after injection of contrast material. However, in cases showing focal hyperostosis, Lee *et al.* suggested that it was possible to predict the origin of inverted papilloma with a high probability [13].

#### 4. Fungal rhinosinusitis

The degree of necrosis and calcification on CT of ANPs raised suspicion regarding fungal infection. In our series, two cases were diagnosed as fungal infection on preoperative CT scan.

The information obtained from the CT scan and MRI, together with the clinical findings, may provide the best guidelines for clinical management. Chronic inflammatory disease is often associated with mucosal thickening and sclerosis of the bone, particularly within the sinuses [14]. Chronic extramucosal fungal sinusitis develops as a saprophytic growth in retained secretions in the sinus cavity. The imaging manifestations of chronic mycotic rhinosinusitis may be nonspecific or highly suggestive of the presence of fungal infection. The presence of diffuse increased attenuation within the paranasal sinuses and nasal cavity should be considered as chronic allergic hypersensitivity aspergillosis [14]. However, bony destruction associated with fungal infection is rare, and ANPs are negative on fungal staining.

#### 5. Malignant tumor

In a relatively uncommon presentation, ANPs may cause extensive bone erosion and remodeling or epistaxis. The presence of any of the latter features can raise the clinical suspicion of a malignant process [1]. In general, ANPs have a long disease history. In the present study as well as in previous reports, ANPs show heterogeneous density, filling the nasal cavity and/or maxillary sinus. The mass shows minimal enhancement on the edge of the lesions. The edge of ANPs on CT is clear and does not invade the peripheral fat layer. However, malignant tumors occur in the elderly and are associated with a relatively short disease history [14]. The bony erosion of malignant tumors is destructive and the edges are indistinct; moreover, the peripheral fat layer is invaded and disappears [15]. On a contrast-CT scan, malignant tumors show heterogeneous enhancement, but hyperostosis and removal of maxillary/nasal cavity walls are rare.

## Materials and Methods

### Patients

From January 1997 to August 2011, 31 ANP cases were treated by surgical excision and were confirmed pathologically at our hospital. The institutional review board of The First Affiliated Hospital, College of Medicine, Zhejiang University approved the present study, and written informed consent was obtained from the patients before inclusion. We reviewed a pathology database and subsequent query of medical records to determine which of these patients underwent CT. We scored nasal endoscopic findings using the Lund–Kennedy (LK) system, which scores the following signs: polyps (0, none; 1, middle meatus; 2, beyond middle meatus; 3, complete obstruction); discharge (0, none; 1, clear and thin; 2, thick and purulent); edema (0, absent; 1, mild; 2, severe); and scarring (0, absent; 1, mild; 2, severe). For our analysis, we summed the scores of the right and left nasal cavities for each sign, resulting in a range of possible scores of 0 to 9 for each sign and a total maximum score of 18. We defined an abnormal endoscopic score as any of 1 or above [5].

### Preoperative CT

CT was performed in all patients using a Toshiba TCT600HQ (Toshiba 16-slice spiral). All patients underwent coronal CT, and six patients received contrast enhancement with Ultravist (300 mg/100 ml) at a dose of 1.5 ml/kg and an injection rate of 2 ml/s, with a scan thickness of 3.2 mm and pitch of 1.0, scanning from the frontal sinus to the bottom of the maxillary sinus; some changes were observed according to the scope of the disease. The images used a soft tissue window and bone window. Using the Lund–Mackay (LM) scale, we scored each sinus (maxillary, frontal, anterior ethmoid, posterior ethmoid, and sphenoid) using the following scale: 0 (no opacification); 1 (partial opacification); and 2 (complete opacification). We scored the ostiomeatal complex as 0 (no opacification) or 2 (opacification). Right and left sides were scored separately, and then summed for a possible score of 0 to 24. We defined an abnormal LM score as any of 1 or above [5].

### Surgery

The lesions were removed from 12 patients via the Caldwell–Luc operation. Three patients underwent lateral rhinotomy, 12 had endoscopic resection of the lesions, and four experienced resection of the mass via the Caldwell–Luc operation combined with endoscopic surgery. Intraoperative pathological diagnosis of fast frozen sections showed benign fibrovascular and necrotic tissue.

### Conclusions

CT of ANPs may demonstrate benign bone changes associated with the lesions and may also reflect the fact that that ANPs do not invade peripheral soft tissue. CT demonstrated these lesions consistently and provided information useful for surgical planning.

The English in this document has been checked by at least two professional editors, both native speakers of English. For a certificate, please see: http://www.textcheck.com/certificate/X9t2QL.
